# Solid Medium Droplet Microarray for Miniaturized Antimicrobial Susceptibility Test

**DOI:** 10.1002/smsc.202500513

**Published:** 2025-11-03

**Authors:** Yuliang Shao, Nikolaj K. Mandsberg, Wenxi Lei, Thomas Schwartz, Pavel A. Levkin, Anna A. Popova

**Affiliations:** ^1^ Institute of Biological and Chemical Systems – Functional Molecular Systems (IBCS‐FMS) Karlsruhe Institute of Technology (KIT) Hermann‐von‐Helmholtz‐Platz 1 76344 Eggenstein‐Leopoldshafen Germany; ^2^ Institute of Functional Interfaces (IFG) Karlsruhe Institute of Technology (KIT) Hermann‐von‐Helmholtz‐Platz 1 76344 Eggenstein‐Leopoldshafen Germany; ^3^ Institute of Organic Chemistry (IOC) Karlsruhe Institute of Technology (KIT) Fritz‐Haber‐Weg 6 76131 Karlsruhe Germany

**Keywords:** antimicrobial susceptibility testing, droplet microarray, high‐throughput, miniaturization, minimum inhibitory concentration

## Abstract

Antimicrobial susceptibility testing (AST) that is easily adaptable for point‐of‐care (POC) use is essential for addressing the growing threat of antibiotic resistance. Here, the solid medium droplet microarray (SM‐DMA), a simple yet versatile testing platform consisting of a single microscope slide patterned with an array of 80 agar droplets (6–8  μL each), containing customizable combinations of clinically relevant antibiotics, is introduced. The test allows for easy manual sample application and features a colorimetric self‐check readout. Using *E. coli* (DSM498) as a model organism, accurate determination of minimum inhibitory concentrations for clinically relevant antibiotics (cefotaxime, ciprofloxacin, and ampicillin), producing results consistent with EUCAST clinical breakpoints, is demonstrated. Furthermore, SM‐DMA facilitates combinatorial antibiotic testing, represented by intuitive viability heatmaps. The platform is more time efficient (≈16–18 h total) compared to the conventional agar plate‐based methods. Owing to the robustness, ease of use, and independence from specialized equipment, the SM‐DMA can be adapted for POC applications by nontrained personnel or even by patients themselves.

## Graphical abstract

The agar‐based miniaturized platform, the Solid Medium Droplet Microarray (SM‐DMA), integrates 80 agar droplets on a microscope slide for rapid, visual antimicrobial susceptibility testing (AST). Combining robustness, simplicity, and minimal material use, SM‐DMA enables point‐of‐care applications and reliable minimum inhibitory concentration (MIC) determination without specialized equipment, offering a practical alternative to conventional AST methods.

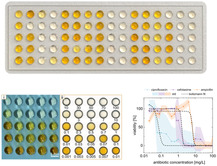

## 1 Introduction

Multidrug‐resistant (MDR) bacteria present a growing challenge to global healthcare, posing serious risks to the patient and straining economic resources. These pathogens have developed mechanisms to render antibiotics ineffective, complicating treatment, especially in immunocompromised populations like young or older people.^[^ ^1^ ^]^ The World Bank estimates that MDR bacteria could reduce annual gross domestic product by 1.1% to 3.8%, with healthcare costs potentially escalating by billions.^[^ ^2^ ^]^ The emergence of new resistances, coupled with the costly, lengthy process of developing new antibiotics, has led even major pharmaceutical companies to retreat from this field, highlighting the urgent need for alternative approaches for mitigation of rapid development of antibiotic resistance.^[^ ^3^, ^4^ ^]^

A significant driver of MDR development is the widespread use of broad‐spectrum antibiotics. From 2000 to 2015, the global antibiotic consumption increased by 65% (21.1 billion to 34.8 billion defined daily doses (DDDs)), with 39% of the total DDDs attributed to broad‐spectrum penicillin in 2015.^[^ ^5^ ^]^ This over‐reliance on broad‐spectrum antibiotics has only worsened during the SARS‐CoV‐2 pandemic, where misuse, overuse, and extended hospital stays have intensified MDR threats.^[^ ^6^ ^]^ The World Health Organization warns that antibiotic resistance is now reaching alarming levels worldwide, with new resistance mechanisms emerging that jeopardize our ability to treat common infections effectively. To mitigate the crisis, the use of antibiotics should be standardized and optimized, ensuring that appropriate antibiotics are used, at the correct doses and for the proper duration.^[^ ^7^, ^8^, ^9^ ^]^

Molecular diagnostic methods offer detection of antibiotic resistance by identifying known specific bacterial species or genetic markers associated with resistance. Techniques such as real‐time polymerase chain reaction (PCR) rapidly identify known resistance markers like carbapenemase genes directly from clinical samples.^[^ ^10^, ^11^ ^]^ Advanced methods like xMAP Technology employ color‐coded microspheres coated with capture molecules for specific detection of target bacteria or resistance genes.^[^ ^12^ ^]^ However, these molecular methods require prior knowledge of biomarkers associated with MDR and do not allow for the identification of unknown resistance species and options for their treatment.

Personalizing antibiotic therapy through experimental antimicrobial susceptibility testing (AST) can reduce unnecessary antibiotic use by determining the minimum inhibitory concentration (MIC) for various antibiotics, allowing precise antibiotic selection and optimal dosing for specific infections. AST methods involve culturing bacteria isolated from patient samples in the presence of antibiotics to assess their sensitivity or resistance. Various methods, both traditional and modern, have been developed for the identification of effective antibiotics for a patient in clinical microbiology laboratories. Traditional techniques include broth microdilution, disk diffusion (Kirby–Bauer method), and gradient diffusion (E‐test). While newer technologies are available, these classic approaches remain widely used due to their proven reliability and standardized protocols.^[^ ^13^ ^]^ However, these methods typically require a full laboratory infrastructure, prolonged incubation times (24–48 h), and substantial manual effort, leading to delays that hinder timely clinical decision‐making.

Emerging automated and miniaturized systems have streamlined traditional methods. Platforms such as the VITEK 2 employ disposable cards containing multiple antibiotic wells to rapidly determine MIC values through bacterial growth comparisons against established standards.^[^ ^14^ ^]^ Similarly, the BD Phoenix system provides real‐time colorimetric detection of bacterial viability, enabling fast AST results.^[^ ^15^ ^]^ The PA‐100 AST system, utilizing nanofluidic and phase contrast microscopy, delivers rapid results within 30 min for five common urinary tract infection‐causing bacteria, *Escherichia coli*, *Klebsiella pneumoniae*, *Proteus mirabilis*, *Enterococcus faecalis*, and *Staphylococcus saprophyticus*.^[^ ^16^ ^]^ In addition, several miniaturized microfluidic systems have been reported. For instance, Boedicker et al. demonstrated that confining single bacteria in nanoliter droplets enables rapid MIC testing through stochastic encapsulation.^[^ ^17^ ^]^ Similarly, Churski et al. developed a droplet‐based gradient generator to perform parallel, miniaturized MIC profiling with precise antibiotic concentration control.^[^ ^18^ ^]^ Despite their advantages, these systems rely on pricey microfluidic pumps and require specialized expertise, which limits their accessibility and ease of use.

Systems enabling point‐of‐care (POC) use are rather limited. One example is the FlexicultSSI‐Urinary Kit, which simplifies testing by directly incubating urine samples on chromogenic agar overnight, allowing for visual assessment within 18–24 h.^[^ ^19^, ^20^ ^]^ Another example, Wat et al. introduced a Self‐Dilution for Faster Antimicrobial Susceptibility Testing (SDFAST) microfluidic chip that integrates antibiotic dilution without the need for microfluidic pumps. This disposable device generates optical readouts for rapid, instrument‐free AST.^[^ ^21^ ^]^

Despite these available technologies, AST is not routinely employed in prescribing antibiotics to the general public due to factors such as infrastructure cost, time delays, and requirement of laboratory infrastructure and personnel.^[^ ^22^, ^23^ ^]^ Here, we introduce the solid medium droplet microarray (SM‐DMA)—a promising platform that addresses current limitations and enables broader implementation of AST. The main novelty of the SM‐DMA system is the use of open DMAs on a hydrophilic–hydrophobic patterned surface with solidified agar droplets to create an off‐the‐shelf, easy‐to‐use POC chip test. Compared to conventional well plates and microfluidic chips, which rely on physical walls or pumps and thus consume more reagents and require specialized equipment, DMAs provide an open, wall‐less format that confines nanoliter‐ to microliter‐sized droplets via wettability patterns. Building on this principle, SM‐DMA employs solidified agar microdroplets instead of liquid droplets, enhancing robustness, minimizing evaporation, and allowing preloaded, shelf‐stable antibiotic arrays. This unique combination makes SM‐DMA a cost‐efficient, user‐friendly, and portable platform ideally suited for POC AST.^[^ ^24^, ^25^ ^]^ The SM‐DMA offers open‐format, robustness, customizable antibiotic loading, isolated test agar droplets, portability, and compatibility with equipment‐free operation by nonspecialist users. These features of SM‐DMA enable accurate antibiotic identification with minimal training, making it ideally suited for POC applications.

In this work, we have successfully fabricated and characterized the SM‐DMA, incubated *E. coli* DSM498 strain, and applied a colorimetric readout method to determine the MIC of three clinically relevant antibiotics, cefotaxime, ciprofloxacin, and ampicillin, and their combinations. The MIC values obtained using SM‐DMA allowed us to accurately classify the *E. coli* DSM498 strain as either susceptible or resistant to the tested antibiotics. This classification was guided by the clinical breakpoints published by the European Committee on Antimicrobial Susceptibility Testing (EUCAST), which provide threshold values for interpreting antimicrobial susceptibility test results. In addition, we demonstrated the potential of SM‐DMA for testing antibiotic combinations and determining the minimal effective dosage of each antibiotic within the combinations. The simplicity, accuracy, and visual readout capability of SM‐DMA position it as a potential POC AST device, enabling rapid, user‐friendly testing outside of clinical laboratories. Therefore, in this study, we have, for the first time, demonstrated the fabrication and application of a solid agar‐based medium for bacterial AST, representing a significant step toward making AST more accessible and widely applicable.

## 2 Results

### 2.1 Concept of SM‐DMA Platform

**Figure 1:**
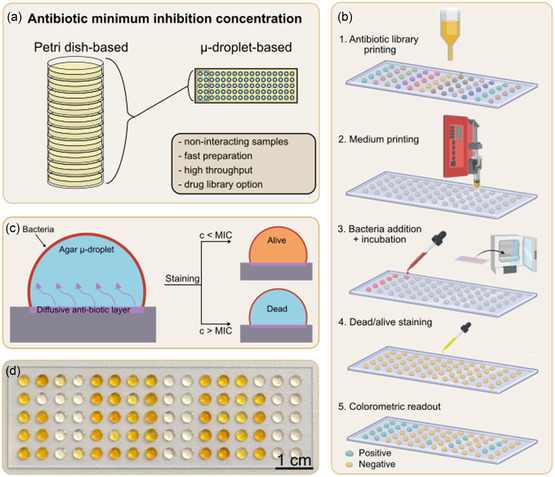
Concept of miniaturized antibiotic screening using SM‐DMA. a) Schematic representation of the downscaling process from agar Petri dishes to an array of agar μ‐droplets, emphasizing the key advantages of reduced culturing volumes. The complete separation of individual agar droplets facilitates high‐throughput screening and enables the exploration of antibiotic combinations. b) Schematic representation of SM‐DMA workflow: (1) printing and drying antibiotic library on a DMA slide, (2) adding agar media to hydrophilic spots and letting them solidify, (3) applying bacterial suspension and incubating overnight, (4) staining with cell counting kit, and (5) colorimetric readout and interpreting the data. c) Schematic representation of a single agar droplet with antibiotic layer dissolving and diffusing into the agar μ‐droplet. Colorimetric viability staining reveals inhibition of bacteria grown in the presence of different concentrations of antibiotics, determining MIC. d) Representative photo of the SM‐DMA containing different concentrations of antibiotics after incubation and staining of bacteria. Dark yellow μ‐droplets indicate the presence of viable bacteria (c < MIC), while transparent μ‐droplets indicate inhibited bacterial growth (c > MIC). The figure was generated using BioRender platform.

A schematic representation of the SM‐DMA concept and the workflow for a miniaturized antibiotic susceptibility test using this platform is presented in **Figure** **1**. With SM‐DMA, we introduce the conceptual shift from traditional Petri dish‐based bacterial screening to the agar μ‐droplet arrays. The SM‐DMA presented here consists of 80 circular hydrophilic spots, each 3 mm in diameter, on a superhydrophobic background. Each hydrophilic spot can confine 6–8 μL droplet of agar. Due to the absence of physical borders between the agar droplets, a high‐density droplet format can be achieved. The superhydrophobic borders ensure complete physical separation of the μ‐droplets, providing contamination‐free conditions between the droplets. Additionally, each μ‐droplet can accommodate a unique combination of antibiotics, media, or samples, enabling diverse and complex screenings. SM‐DMA represents a significant advancement over traditional 10 cm agar Petri dishes. The total agar volume required for the entire slide is ≈480–640 μL, accommodating 80 distinct conditions. This translates to about 30 μL per antibiotic across 5 concentrations, compared to 5 mL per antibiotic when using a Petri dish, a reduction by a factor of about 167. Consequently, the amount of each antibiotic needed for testing is significantly reduced in proportion to the smaller culturing volume. In addition to minimizing the use of consumables, the array format of SM‐DMA allows each of the 80 spots to accommodate different conditions or even distinct patient samples. As few as three spots (including the control) are sufficient to determine the susceptibility of a specific bacterium to a particular antibiotic. In contrast, traditional Petri dishes require all conditions to share a single agar surface. This flexibility makes SM‐DMA particularly advantageous for designing versatile and efficient screening experiments.

The detailed overview of the workflow of SM‐DMA preparation and screening process is shown in Figure 1b. In step 1, the antibiotic solution is dispensed onto the DMA slide using a noncontact low‐volume dispenser and dried at room temperature. In step 2, droplets of heated agar media are deposited onto the hydrophilic spots using a pump‐controlled syringe and allowed to cool at room temperature until the agar droplets solidify. As depicted in Figure 1c, the preprinted antibiotic layer beneath each μ‐droplet diffuses into the warm agar medium. Next (step 3), bacterial solution is applied onto the solidified agar droplets containing antibiotics and incubated overnight at 37 °C. In step 4, bacteria are stained with a formazan salt‐based viability assay (Kit‐8), which provides a colorimetric readout of bacterial viability (absorption peak at 450 nm,^[^ ^26^ ^]^ appearing yellow), enabling MIC determination. In the final step (step 5), the SM‐DMA is scanned using a conventional photo scanner, and the color differences are analyzed to differentiate between bacterial growth (dark yellow) and inhibition (transparent). Notably, steps 1 and 2 can be completed in advance, creating a ready‐to‐use antibiotic library that potentially can be used as off‐shelf POC AST and MIC determination test. Determining the inhibition of bacterial growth, which reflects susceptibility or resistance, can be done visually as a simple yes/no answer. This makes the test potentially suitable for home use by untrained personnel.

**Figure 2:**
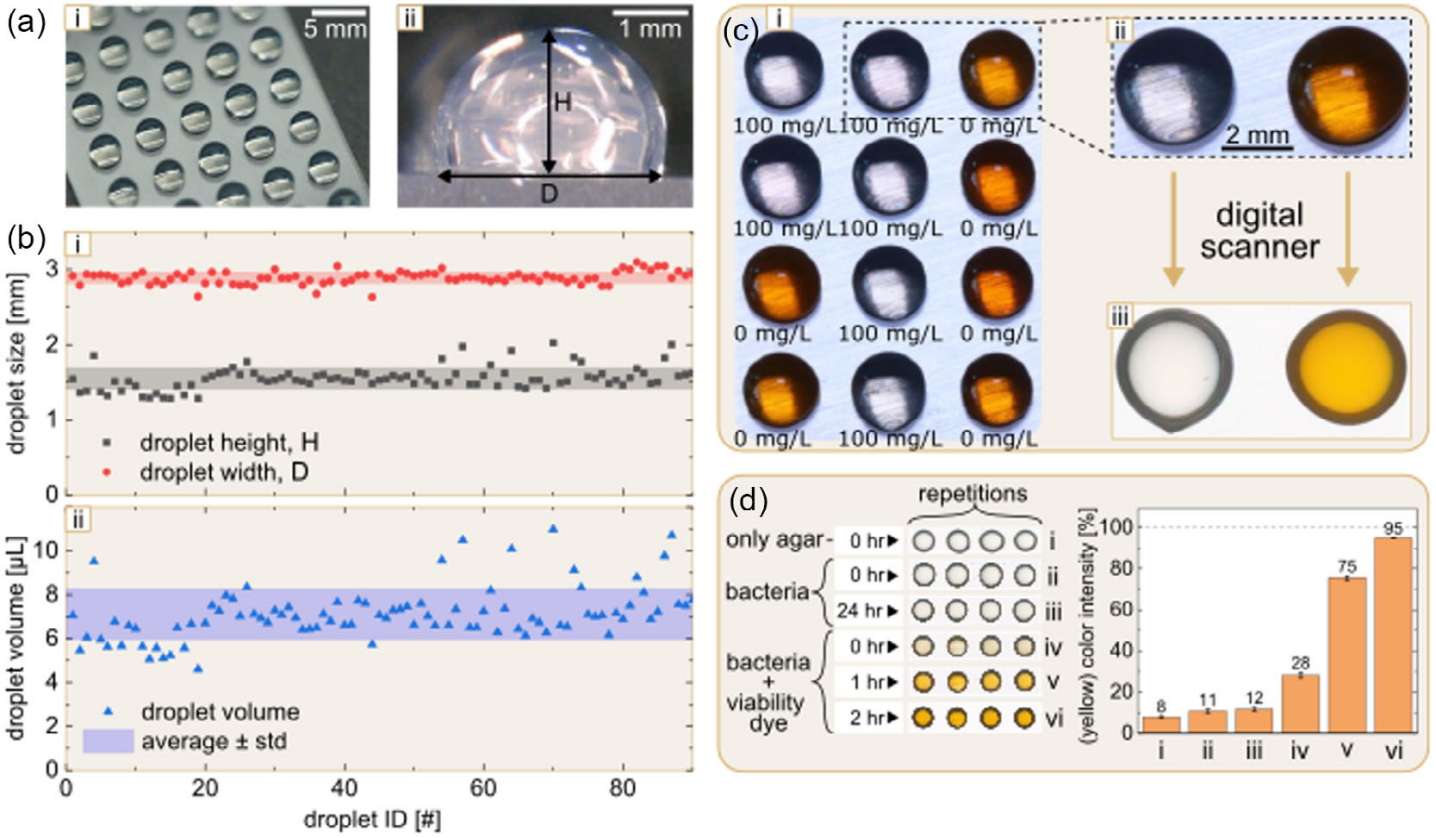
Characterization and validation of SM‐DMA platform. a) Photographs of agar array (left, angled view) and single μ‐droplet (right, side view). b) The graphs showing the distribution (*n* = 90) of heights and diameters (i) and volumes (ii) of agar droplets formed on SM‐DMA measured across 90 μ‐droplets. c) (i) Photograph of a 4 × 3 array of SM‐DMA agar droplets containing *E. coli* and different concentrations of cefotaxime, following staining with Kit‐8 viability dye. The cefotaxime concentration is indicated for each droplet. (ii) Zoom into two droplets and (iii) the same droplets scanned by a photo scanner. d) Validation of bacterial culture and staining on agar μ‐droplets, with quantification of colorimetric digital scans of SM‐DMA. (i) Digital scans of SD‐DMA at different stages of workflow: (i) only agar, (ii–iii) agar with bacteria (incubated for 0 or 24 h), and (iv–vi) agar with bacteria after 24 h of growth stained with Kit‐8 dye for 0, 1, and 2 h. Bars represent mean values ± SD (*n* = 3).

Figure 1d shows the photo 80‐spot SM‐DMA after incubation of *E. coli* (DSM498) with various concentrations of cefotaxime. Dark yellow μ‐droplets indicate viable bacteria incubated with cefotaxime at concentrations below the MIC, while transparent μ‐droplets indicate inhibition of bacterial growth by the antibiotic at concentrations above the MIC. The color change on SD‐DMA is analyzed by ImageJ, assigning the numeric value reflecting the intensity of the color change to each spot. At each stage, the intensity of yellow color is estimated using Equation (2) to determine **Figure** **2**d(i–iii) background signals and Figure 2d(iv–vi) colorimetric variations due to staining after different incubation times. These values are then normalized to the control and plotted against the concentrations of a drug. Later, we use the Boltzmann fitting to create a dose–response curve, from which we define the IC90, the concentration of an antibiotic required to inhibit the growth of 90% of a given bacterial population. Since the MIC is defined as the lowest concentration of an antibiotic that inhibits bacterial growth, we approximate the IC90 as equivalent to the MIC in the SD‐DMA assay. Alternatively, MIC can be determined from SM‐DMA by analyzing the colorimetric image by the eye and defining the concentration at which the growth is inhibited, which is indicated by the absence of yellow staining. The obtained MIC values are then compared to established clinical breakpoint values published by EUCAST. Specifically, we refer to the MIC (R), which represents the threshold concentration of an antibiotic required to inhibit the growth of susceptible bacteria. In other words, if bacteria survive at a concentration higher than this threshold, they are considered resistant. Based on this comparison, the bacterial strain is categorized as either susceptible or resistant to the tested antibiotics. Thus, SM‐DMA assay can be utilized to determine MIC and therefore susceptibility or resistance of a given bacterial strain or patient samples according to EUCAST standards.

### 2.2 Fabrication and Characterization of SM‐DMA

Precise identification of the MIC of antibiotics is contingent upon the robustness and reproducibility of the SM‐DMA fabrication process. It is crucial to ensure that agar droplets have uniform volume both within a single SM‐DMA and between different DMAs, as the final antibiotic concentration depends on the droplet volume (Figure 2a). The agar solution is highly viscous and solidifies within a temperature range of 34–43 °C. Therefore, droplet formation needed to occur at temperatures exceeding this range. To ensure smooth and consistent deposition of agar μ‐droplets, the agar medium was heated to 60 °C and loaded into a syringe with a diameter of 21.5 mm. A syringe pump was employed to produce a continuous flow of the agar solution at a rate of 5 μL s^−1^. The agar droplets were actively captured using a DMA. This process was performed manually by hand, with the operator carefully controlling the capture of each droplet onto the hydrophilic spot surface. The fabrication of a single SM‐DMA containing 80 spots required ≈3 min.

We have characterized volume homogeneity of the agar μ‐droplets using side‐view images obtained with a Keyence 3D VHX‐5000 microscope. ImageJ software was used to measure the height (*h*) and diameter (*d*), of each droplet (Figure 2b(ii)). Assuming that the droplet has a shape of a spherical cap, the corresponding volumes (*V*), presented in Figure 2b(i), were calculated using Equation (1).^[^ ^27^ ^]^ Inspecting 90 droplets across three different DMA slides, the average volume was 7.1 ± 1.2 μL (avg ± std); 68% of the droplets had volumes between 6.2 and 7.8 μL.

(1) $$V=1/24\cdot \pi \cdot h\left(3d^{2}+h^{2}\right)$$

### 2.3 Colorimetric Readout and Analysis on SM‐DMA

It is important to develop a simple method for estimating the effect of tested antibiotics on SD‐DMA. To achieve this, we established a colorimetric viability assay that enables single‐step bacterial growth assessment without the need for expensive laboratory equipment. Specifically, after overnight cultivation of the bacterial sample on SM‐DMA, cell counting Kit‐8 (WST‐8 salt) was dispensed onto the agar droplet, and after 2 h of incubation, a scanned image was acquired using the photo scanner. Figure 2c shows clear difference in color between the agar droplet containing high concentration of the antibiotic cefotaxime c > MIC, which causes inhibition of the growth of *E. coli* (transparent droplets), and droplets containing low concentration of the antibiotic cefotaxime c < MIC, where bacterial growth was not inhibited (dark yellow droplets).

In order to validate the colorimetric method and demonstrate that the change from transparent to yellow color is caused by the living bacteria and not experimental artefacts, we compared the color change during six different stages of the protocol. The representative scans of four μ‐droplets at each of the six stages are shown in Figure 2d‐left: (i) freshly made SM‐DMA agar droplets, (ii and iii) SM‐DMA with *E. coli* bacteria after 0 h (ii) and 24 h (iii) of incubation without addition of Kit‐8, and (iv–vi) SM‐DMA with *E. coli* bacteria after 24 h of incubation followed by addition of Kit‐8 for ≈0 h (iv) 1 h (v), and 2 h (vi). These images clearly demonstrate that the color change is caused by bacterial growth and not by other factors.

An active component of Kit‐8 viability dye tetrazolium salt WST‐8 is reduced by live bacteria dehydrogenases to a water‐soluble formazan dye of a yellow–orange color that can be seen in the images. The formazan dye absorbs light with a wavelength of 450 nm (blue light).^[^ ^26^ ^]^ This adsorption is proportional to the concentration of live bacteria and used to quantify bacterial growth.^[^ ^28^ ^]^ In order to quantify bacterial growth on SM‐DMA, we had to convert the color to a numeric value. Under the red, green, and blue (RGB) additive color model, a color is composed of RGB channels;^[^ ^29^ ^]^ therefore, we have measured the RGB composition of each agar droplet. Afterward, we have quantified the intensity of the yellow color as follows

(2) $$I_{Y}=\left(1-\frac{I_{B}}{255}\right)\times 100\%$$

where *I*_Y_ is the intensity of yellow color of the agar droplets on SM‐DMA and *I*_B_ is the blue light intensity from the RGB analysis. By using this method, we have quantified the intensity of yellow color on SM‐DMA scans presented in Figure 2d‐left (Figure 2d‐right). The intensity value of Luria–Bertani (LB) agar without bacteria and bacteria without Kit‐8 dye is significantly lower compared to the intensity observed in live bacteria with the addition of Kit‐8 dye. Additionally, there is a clear trend of increasing intensity with longer incubation times with the dye (Figure 2d‐right), indicating that the method functions effectively. In order to compare the color intensity between the different concentrations of antibiotics and reflect the growth rate and number of living bacterial cells in these experimental conditions, we used the following equation

(3) $$V_{b}=\left(1-\frac{I_{B}-I_{B,c\;=0}}{I_{\text{B,background}}-I_{B,c\;=0}}\right)\times 100\%$$

where *V*_b_ is the sample viability, *I*_B_ is the sample's blue intensity, *I*_B,*c*=0_ is the average blue intensity of stained agar droplets containing bacteria and no antibiotics, and *I*_B,background_ is the blue intensity of the superhydrophobic background.

### 2.4 AST on SM‐DMA

**Figure 3:**
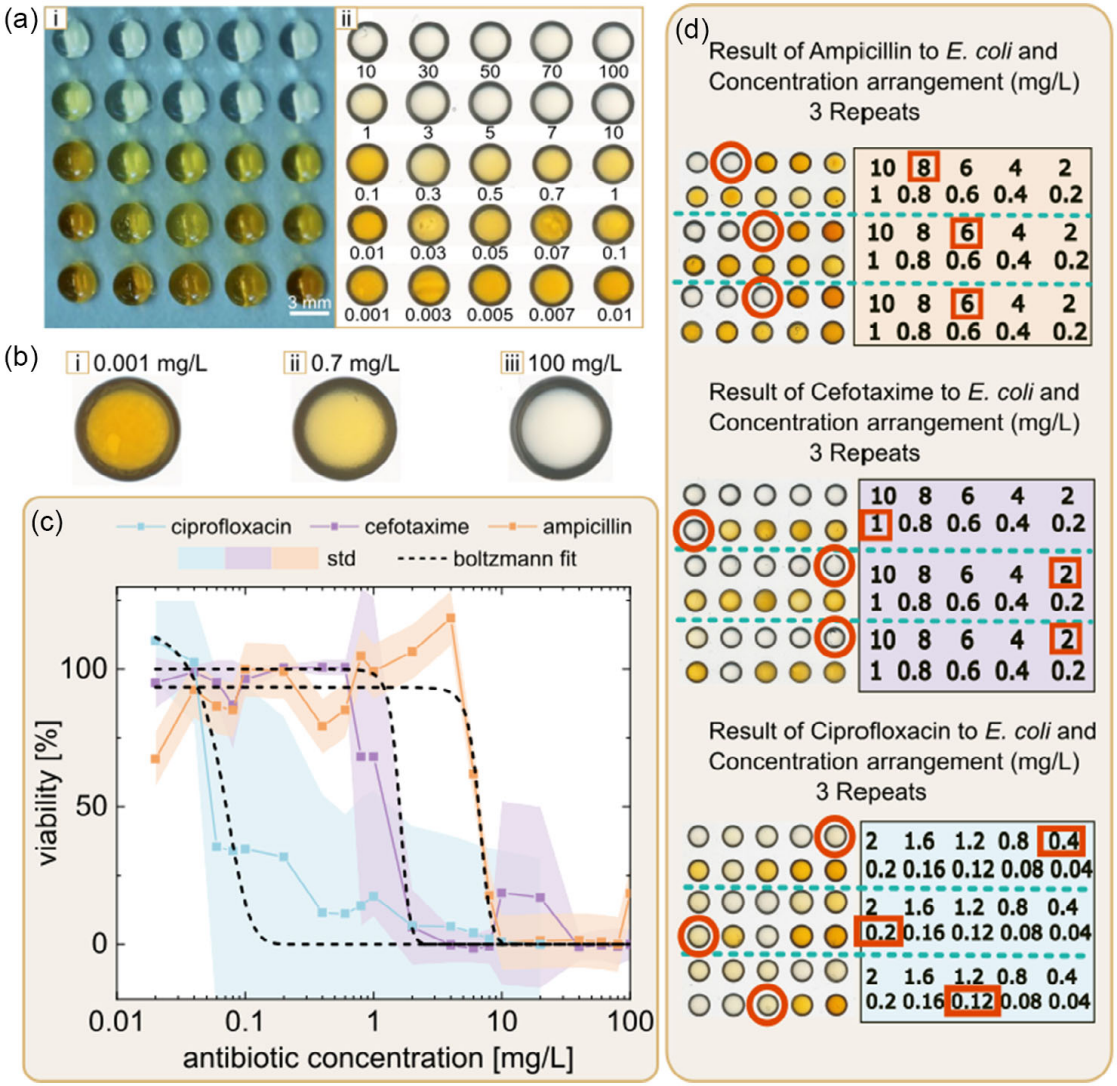
Determination of the MIC using SM‐DMA. a) The photograph (i) and the scan (ii) of 5 × 5 droplet SM‐DMA containing different concentrations of cefotaxime (ranging from 0.001  to 100 mg L^−1^) after incubation of *E. coli* and Kit‐8 staining. b) Zoom‐in on three agar droplets containing 0.001 mg L^−1^ (i), 0.7 mg L^−1^ (ii), and 100 mg L^−1^ (iii) of cefotaxime. The figure demonstrates (i) noninhibited, (ii) partially inhibited, and (iii) fully inhibited growth of bacteria. c) The graph showing the response curve to three antibiotics, i.e., ciprofloxacin, cefotaxime, and ampicillin (*n* = 3). The curve was fitted with a Boltzmann function to determine the corresponding MIC. d) The colorimetric readout from AST. The blue lines separate three parallel experiments for each antibiotic. The MIC for this experiment group is marked by red circles. These circles represent critical points identified through visual inspection. Orange rectangles show the antibiotic concentration corresponding to each circled MIC point. Dots represent mean values; shaded areas denote ± SD from three independent experiments (*n* = 3).

Having established and validated the SM‐DMA workflow, we then used it for determination of MIC of cefotaxime, which is commonly used in clinics to treat *E. coli* (DSM498) infections (**Figure** **3**). For this, we have used 25 different concentrations of antibiotic, in 3 repetitions. Figure 3a,b illustrates the colorimetric readout on SM‐DMA after staining of bacteria treated with a range of concentration of antibiotic cefotaxime. The results can be divided into three groups. The first group consists of agar droplets with an antibiotic concentration below 0.3 mg L^−1^ (exemplified in Figure 3b(i)). Here, the color of the agar droplets is dark yellow, indicating that *E. coli* growth is not inhibited at these concentrations of antibiotic. The second group of agar droplets contained higher concentrations of the antibiotic cefotaxime, from 0.3 to 1 mg L^−1^ (exemplified in Figure 3b(ii)). The resulting color of this group is pale yellow, indicating that the growth of *E. coli* is partially inhibited. The third group of droplets with the highest concentrations of cefotaxime showed that the droplets are clearly transparent as the growth of *E. coli* is fully inhibited (Figure 3b(iii)). Through visual examination of stained SM‐DMA, we could identify that the cefotaxime concentration inhibiting *E. coli* growth ranged between 0.3 and 3 mg L^−1^. This range corresponds to the MIC of cefotaxime and aligns with the clinical breakpoints and dosage recommendations provided by EUCAST, which is 1 mg L^−1^. These findings demonstrate the feasibility of visually determining the MIC using SM‐DMA.

To further validate the protocol, we have used it for determining MIC for three antibiotics, ampicillin (MIC ≈8 mg L^−1^, EUCAST), cefotaxime (MIC ≈1 mg L^−1^, EUCAST), and ciprofloxacin (MIC ≈0.125 mg L^−1^, EUCAST), and tested in 10 different concentrations and in 3 independent repeats. These are conventional drugs that are commonly prescribed, with MICs spanning two orders of magnitude, ranging approximately from 0.1 to 8 mg L^−1^. Figure 3c(i) shows the dose–response curves for all three tested antibiotics. The response curves exhibit a sigmoidal shape and were fitted using a Boltzmann sigmoidal function (Equation (4))

(4) $$V_{c}=\frac{V_{c\text{>>}c_{0}}}{1+e^{-k\left(c-c_{0}\right)}}$$

where *c* represents the antibiotic concentration, *c*_0_ is the estimated IC50, *k* is the transition coefficient and is proportional to the slope at IC50, and $V_{c>>c_{0}}$ is the viability for concentration much larger than IC50, accounting for experimental uncertainty. Utilizing the dose–response curves, we determined the IC90 values, which in our study correspond to the MIC. These values represent the antibiotic concentration required to inhibit 90% of bacterial growth, as interpolated from the fitted dose–response curve (**Table** **1**).

**Table 1:** The result from Boltzmann fitting and colorimetric method compared to MIC breakpoint published by EUCAST.

Antibiotic	MIC result from Boltzmann fitting [mg L^−1^]	MIC result from colorimetric readout [mg L^−1^]	MIC (R) breakpoint (EUCAST) [mg L^−1^]
Ampicillin	8.22	6–8	8
Cefotaxime	1.9	1–2	2
Ciprofloxacin	0.1	0.12–0.4	0.5

As SM‐DMA readout is based on colorimetric readout that can be visually analyzed, MIC of antibiotics can be determined without scanning, image analysis, and mathematical functions, but by simply visual examination. In other words, the user can estimate MIC just looking at the SM‐DMA. It is particularly suited for point‐of‐care testing and can be used by untrained personnel, including patients, without the need for specialized equipment, even in home environments. Figure 3d demonstrates three repeats of AST tests for cefotaxime, ciprofloxacin, and ampicillin on *E. coli* DSM498. Orange rectangles highlight the MIC points that can be visually identified on droplets where the yellow color begins to fade compared to neighboring droplets with lower concentrations of antibiotics, indicating the onset of bacterial growth inhibition. Table 1 contains the ranges of these concentrations. As could be seen, by visual examination, we could determine the same range of MIC by using full analysis, including image analysis and mathematical fitting.

Table 1 compares the MIC values obtained by both the Boltzmann fitting approach and visual examination of stained SM‐DMA, with the published EUCAST MIC values. Both methods gave the MIC values close to the standard breakpoint concentrations of these three antibiotics against Enterobacteria, to which *E. coli* belong to. According to the identified MIC using SM‐DMA, *E. coli* DSM498 strain was identified as being susceptible to all three antibiotics, cefotaxime, ampicillin, and ciprofloxacin, because the identified MIC is lower or equal to EUCAST MIC.^[^ ^30^ ^]^

Thereby, we have proved that SM‐DMA is capable of detecting the MIC value of cefotaxime, ampicillin, and ciprofloxacin on *E. coli* DMA498 and arrived to a conclusion that *E. coli* DSM498 is susceptible to all three tested antibiotics according to the clinical breakpoint published by EUCAST.

**Figure 4:**
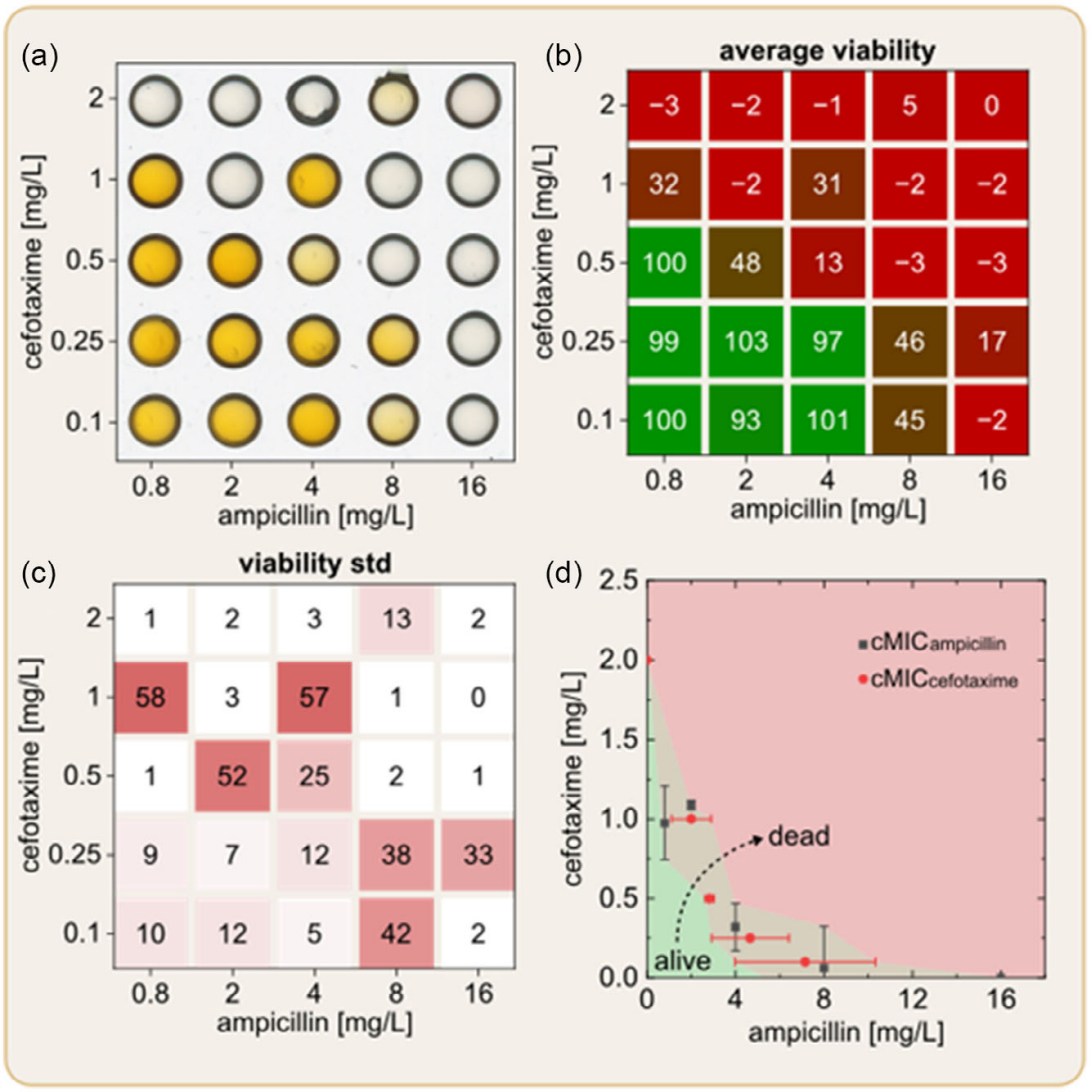
Combinatorial cMIC test. a) Digital scans of the stained SM‐DMA. b) Heatmap showing average viability of *E. coli* treated with ampicillin and cefotaxime and their combinations (*n* = 3). c) Heatmap of corresponding SDs for the inhibition data. d) Phase diagram displaying the combinatorial MIC (cMIC) values. The cMIC values were extracted from the viability heatmaps in (b) by fitting each row and column with Boltzmann curves to model dose–response relationships. Data are presented as mean ± SD, with *n* = 3 independent experiments; error bars indicate SD.

The miniaturization of antibiotic experiments significantly increases the number of individual experimental conditions that can be conducted simultaneously, offering greater flexibility in customizing the AST. This makes the SM‐DMA platform particularly suitable for combinatorial MIC testing, where multiple drugs are combined in a single agar droplet. Such testing more closely mimics real‐world scenarios, as patients are often treated with drug combinations. Typically, performing combinatorial investigations on traditional platforms is impractical due to the high consumption of materials and space, but the miniaturized SM‐DMA platform overcomes these challenges. As a proof of concept, we tested 25 combinations of cefotaxime and ampicillin, with each drug spanning a 20‐fold concentration range (cefotaxime from 0.1  to 2 mg L^−1^ and ampicillin from 0.8 to 16 mg L^−1^). The representative digital scan of one of the three repeats of SM‐DMA after the combinatorial screening is presented in **Figure** **4**a. We analyzed the color changes after staining and quantified them into numeric values, following the method previously described using Equation (4). Figure 4b shows the resulting heatmap, containing the viability data averaged from three independent experiments. It illustrates the transition from high viability (lower left corner at low/low concentrations) to inhibition of growth (upper right corner at high/high concentrations). A corresponding standard deviation (SD) heatmap (derived from all three experimental repetitions) demonstrates minimal SD for both low and high concentrations of antibiotics, while a larger SD for the transition concentrations near the IC50 curve (Figure 4b). The substantial SD in viability observed at intermediate concentrations is caused by sensitivity in biological response coupled with potential variations in printing volumes and inherent biological variability between experiments.

Similar to MIC determination for single antibiotics (Figure 3d), we identified MIC values for a combination of two antibiotics, cMIC_ampicillin_ and cMIC_cefotaxime_. cMIC_ampicillin_ was defined as the concentration of ampicillin that has to be added to the fixed concentrations of cefotaxime (0.1, 0.25, 0.5, 1, and 2) in order to inhibit the growth of bacteria (Figure 4d, red). Identically, cMIC_cefotaxime_ was defined as the concentration of cefotaxime that has to be added to the fixed concentrations of ampicillin (0.8, 2, 4, 8, and 16) in order to inhibit the growth of bacteria (Figure 4d, gray). Using cMIC_ampicillin_ and cMIC_cefotaxime_, we have created phase diagram defining three areas of combinations of concentrations of two antibiotics. The middle area depicted in light green includes cMIC of antibiotic combinations. We defined it by connecting the lower and higher bars of SD from both cMIC_ampicillin_ and cMIC_cefotaxime_. The bright green area defines the combination of ampicillin and cefotaxime that do not inhibit the growth of bacteria, while the red area represents the concentrations that has inhibitory effect on the growth of bacteria. By looking at this diagram, a potential clinician can identify which minimal concentration of each antibiotic in their combination is sufficient to inhibit the growth of present bacteria in the tested sample.

## 3 Discussion

In this study, we successfully developed SM‐DMA platform, an array of agar droplets on a surface‐modified glass slide (SM‐DMA). We demonstrated that this array can be effectively used for bacterial culture. Furthermore, we established its utility in conducting both single‐drug dose–response and multidrug dose–response experiments. This innovative approach provides a versatile and cost‐effective platform for microbial studies and drug screening applications that potentially can be adopted as POC test.

SM‐DMA holds 80 homogeneous antibiotic‐containing agar μ‐droplets on a 75 × 25 mm chemically functionalized microscope glass side^[^ ^31^, ^32^ ^]^ effectively replacing 80 Petri dishes while reducing the culturing volume 1000‐fold and consolidating 80 parallel experiments on a single chip. In contrast to previous applications of DMA, which typically use liquid media to culture bacteria or eukaryotic cells for antibiotic discovery or oncology‐related drug sensitivity testing^[^ ^33^, ^34^, ^35^, ^36^, ^37^ ^]^, our SM‐DMA platform for the first time employs solid agar droplets. The use of agar offers multiple advantages: it is a well‐established and selective medium for bacterial cultivation. Incorporating agar into the SM‐DMA platform enhances the stability, packaging, and storage of ready‐to‐use chips, enables simple manual sample application, minimizes evaporation, and prevents cross‐contamination of antibiotics during sample loading.

The SM‐DMA platform presented here contains 6–8 μL agar droplets. However, the platform has significant potential for even further miniaturization of culturing volumes, at least down to 1 μL and less, which would decrease consumption of antibiotics and reagents even further and increase throughput. Throughput is important for testing more antibiotics and their combinations or increasing the number of repeats, thereby enhancing replication and ultimately improving the statistical significance of the results. To achieve this, automating the production of SM‐DMA, as opposed to the manual manufacturing described in this work, would be necessary. This would also help minimize deviations in agar droplet volumes, improving consistency and reproducibility. Alternative approaches to deposit 3D pads in hydrophilic spots on DMA include 1) utilizing 3D printing technology and 2) synthesizing substrates directly on the platform. These strategies also pave the way for incorporating a wider range of materials and expanding the potential applications of the SM‐DMA platform as an array of 3D scaffold materials on a flat surface.

The total time required for SM‐DMA‐based AST is ≈16–18 h. It consists of overnight incubation (≈16 h) followed by a simple 1–2 h viability staining and readout step. Although the method is demonstrated here using pure bacterial strains in suspension, it can also be applied directly to unpurified fluid samples from patients. In such cases, selective agar media can be used to promote the growth of potentially pathogenic bacteria. The SM‐DMA format allows simultaneous testing of multiple antibiotics and samples on a single slide, making it highly efficient for comparative screening. Moreover, the antibiotic panel can be easily customized to include specific drugs of interest, enabling rapid adaptation to clinical needs or local resistance profiles.

This study represents the first successful application of solid medium—specifically agar—for AST on a DMA platform. The use of solid medium introduces several distinct advantages over traditional liquid‐based microdroplet systems. Agar provides mechanical stability and robustness, reducing the risk of droplet evaporation, merging, or accidental disruption during handling. This enhances the reproducibility and reliability of the assay. The solidified format also enables the creation of preloaded, shelf‐stable AST arrays that can be prepared in advance and stored for later use—facilitating POC applications. What is more, it simplifies the assay workflow, allowing for easier sample application without the need for specialized microfluidic equipment. Finally, the portability of the solid medium format makes it suitable for decentralized testing environments, including low‐resource settings and potentially even home‐based use, thus expanding the accessibility of AST beyond centralized laboratories.

In the SM‐DMA workflow, antibiotics such as ampicillin, cefotaxime, and ciprofloxacin are exposed briefly to molten agar at ≈60 °C during droplet deposition. These antibiotics are not super thermal sensitive,^[^ ^38^, ^39^, ^40^, ^41^, ^42^, ^43^, ^44^, ^45^ ^]^ and our experimental results confirm that their antimicrobial efficacy remains unaffected, as evidenced by the MIC values closely aligning with EUCAST clinical breakpoints. While an alternative approach could involve directly dispensing antibiotic solutions onto presolidified agar droplets—allowing passive absorption—our method involves first depositing and drying the antibiotic on the DMA slide before adding the hot agar. This strategy not only ensures even distribution through diffusion but also enables the creation of a stable, preloaded antibiotic library. Such prefabricated SM‐DMA slides offer improved long‐term storage potential, making them suitable for decentralized or household diagnostic applications where ready‐to‐use formats are essential.

We have demonstrated that the determination of the MIC breakpoint using SM‐DMA is reliable, as it aligns with data published by EUCAST. However, we observed some variability in bacterial growth, particularly at antibiotic concentrations near the breakpoint. This variability is expected, as bacterial growth at concentrations close to the MIC can fluctuate due to slight differences in bacterial susceptibility, stochastic effects in cell survival, and potential adaptive responses, leading to heterogeneous outcomes.^[^ ^46^ ^]^ MIC results exhibit inherent variability due to interassay fluctuations and operator‐dependent factors, even under standardized laboratory conditions and with standard protocol. For example, an MIC of 1.0 mg L^−1^ is generally interpreted in a range of 0.5–2.0 mg L^−1^, and values within this twofold range are considered consistent. Awareness of this range of variability is essential for meaningful and accurate interpretation of MIC results.^[^ ^47^, ^48^ ^]^

The choice of the readout assay is another critical factor in ensuring MIC accuracy. In the SM‐DMA antibiotic test, bacterial viability is indicated by a colorimetric shift from clear to yellow, resulting from the conversion of the transparent WST‐8 tetrazolium salt into a yellow formazan dye by living bacteria. This dye is well suited for the current application and easily recognizable by the naked eye. It should be kept in mind that it is possible that other types of agar media can interfere with the stain and affect the clarity of the color interpretation. Other staining solution, for example, resazurin, a commonly used stain, is unsuitable for this system because it reacts with Chromocult Coliform Agar, causing a color change even in the absence of live bacteria. This problem highlights the need for stains that are compatible with a wide range of agar media. Future research could focus on identifying or modifying stains that provide reliable color differentiation across different media, thereby increasing the robustness of the SM‐DMA platform.

Moving forward to improving the SM‐DMA platform in the future, it is important that it has the ability to handle mixed bacterial cultures. Enhancing its capability to target specific bacteria within complex samples could be achieved by developing selective media that support the growth of the species of interest, such as pathogenic bacteria. This approach would require prior knowledge of the bacterial strains likely to be present in the sample to design effective selective media. By enabling precise isolation of target bacteria, this advancement would make SM‐DMA particularly valuable for testing patient samples, which contain both pathogenic and commensal bacteria, ensuring that the test identifies antibiotics effective against the harmful strain. Additionally, expanding the platform's compatibility with more complex clinical and environmental samples would further broaden its potential applications.

We envision SM‐DMA as a ready‐to‐use, user‐friendly, and rapid AST tool, capable of predicting a patient's susceptibility to specific antibiotics. Its solid‐medium format enhances stability and ease of use, making it suitable not only for laboratory settings but also for potential home applications. The SM‐DMA chip, preloaded with locally available antibiotics, allows for simple sample application via pipetting or manual spreading, followed by staining to visually determine the MIC. This straightforward process enables individuals without formal laboratory training or specialized equipment to perform the test, making AST accessible even outside clinical environments. By arranging antibiotics at concentrations aligned with EUCAST‐published MIC breakpoints, SM‐DMA can effectively classify bacterial strains as susceptible or resistant, unlocking exciting potential for decentralized and personalized antibiotic testing.

## 4 Conclusion

In this study, we presented a novel platform, SM‐DMA, for AST. This platform capitalizes on an array of physically isolated agar μ‐droplets on a planar surface, enabling miniaturized and multiplexed antibiotic testing, significantly reducing material consumption and increasing throughput compared to conventional methods.

We have demonstrated the ability of SM‐DMA to quantify bacterial growth, determine MICs of antibiotics, and facilitate combinatorial antibiotic testing. The platform's compatibility with colorimetric viability staining, combined with the accuracy of MIC determination, makes it ideal for rapid POC applications. In addition, the ability to preload the SM‐DMA with an antibiotic library offers potential as a ready‐to‐use device.

Its potential for further miniaturization and automation also suggests a wide range of applications from clinical diagnostics to food safety and wastewater management. Ultimately, SM‐DMA provides a portable, user‐friendly solution for rapid and accurate AST, making it a valuable tool in the fight against antibiotic resistance.

## 5 Experimental Section

5.1

5.1.1

##### Reagents and Equipment

DMAs, patterned superhydrophobic–hydrophilic glass slides (7.5 × 2.5 cm), were obtained from Aquarray GmbH (Eggenstein‐Leopoldshafen, Germany). Each DMA had 80 (16 × 5) circle‐shaped hydrophilic spots (diameter = 2828 μm)^[^ ^49^ ^]^ with distance between each spot 1500 μm. Cefotaxime and ampicillin were purchased from Sigma–Aldrich (Munich, Germany), and ciprofloxacin was purchased from Thermo Scientific (Waltham, Massachusetts, USA). LB agar media was purchased from Roth with composition: tryptone 10 g L^−1^, yeast extract 5 g L^−1^, sodium chloride (NaCl) 10 g L^−1^, and pH value 7.0 ± 0.2. Cell counting KIT‐8 (Dojindo, EU) was used as the staining agent to show the proliferation of the bacterial colony on the SM‐DMA. Phosphate‐buffered saline was used as buffer solution for serial dilutions and prepared according to the following procedure: 0.8 g NaCl (Fisher Scientific, Germany), 0.2 g KCl (Sigma–Aldrich, Germany), 1.44 g Na_2_HPO_4_, (Sigma‐Aldrich, Germany), and 0.24 g KH_2_PO_4_ (Carl Roth, Germany) were dissolved in 800 mL of water and pH value was adjusted to 7.4 with 37% HCl (Carl Roth, Germany), and then, the solution was diluted with water up to 1 L and autoclaved. Keyence VHX‐5000 (Keyence, Germany) was used to image the agar droplets from the side, and CanoScan 8800 F (Canon, Germany) was used to acquire a digital image of the whole SM‐DMA slide. A syringe pump (NE‐300, New Era Pump Systems Inc., USA) was used for agar dispensing, and a spectrophotometer (U‐5100, Hitachi High‐Tech Corporation, Japan) was employed for absorbance measurements.

##### Fabrication of SM‐DMA: Antibiotics

Antibiotics in saline buffer were manually pipetted onto empty hydrophilic spots of DMA prior to agar droplet formation, followed by drying slides at room temperature. Concentrations of antibiotics were adjusted as depicted in **Table** **2**.

**Table 2:** The concentration arrangement of the antibiotics on DMA.

Name of the antibiotic	Stock centration [mg L^−1^]	Volume added to DMA [μL]	Final concentration in agar droplets [mg L^−1^]
Ampicillin	100	10/8/6/4/2	100/80/60/40/20
Ampicillin	10	10/8/6/4/2	10/8/6/4/2
Ampicillin	1	10/8/6/4/2	1/0.8/0.6/0.4/0.2
Cefotaxime	100	10/8/6/4/2	100/80/60/40/20
Cefotaxime	10	10/8/6/4/2	10/8/6/4/2
Cefotaxime	1	10/8/6/4/2	1/0.8/0.6/0.4/0.2
Ciprofloxacin	20	10/8/6/4/2	20/16/12/8/4
Ciprofloxacin	2	10/8/6/4/2	2/1.6/1.2/0.8/0.4
Ciprofloxacin	0.2	10/8/6/4/2	0.2/0.16/0.12/0.08/0.04

##### Fabrication of SM‐DMA: Agar Droplet Deposition and Characterization

Agar droplets were deposited onto DMA containing antibiotics using syringe pump with automated and adjustable flow rate. We have used a syringe with 5 mm diameter and flow rate of 16 μL min^−1^. To prevent solidification of agar during the deposition, hot liquid agar inside syringe was kept above 50 °C by using heat gun during the procedure. The procedure of SM‐DMA fabrication was performed inside closed transparent chamber made out of polymethyl methacrylate material (Plexiglass). The volume extracted per second was calculated as follows

(5) $$V_{r}={\left(\frac{21.5\;\text{mm}}{5\;\text{mm}}\right)}^{2}V_{s}=18.49\cdot 16\frac{\text{μL}}{\text{min}}=295.84\frac{\text{μL}}{\text{min}}\approx 4.93\frac{\text{μL}}{s}$$

where *V*_r_ is the calculated flow rate and *V*_s_ is the flow rate for syringe pump.

In order to achieve the volume of agar droplets of about 10 μL, the DMA slide was manually positioned with hydrophilic spot under the syringe pump for 2 s and then moved to the next hydrophilic spot. For storage and bacterial culture, SM‐DMA was placed inside a 10 cm Petri dish with a humidifying lid containing a tissue wetted with saline buffer; Petri dish was sealed with parafilm for storage and incubation to prevent evaporation.

In order to characterize the homogeneity of fabricated agar droplets on SM‐DMA, images of the droplets from the side were acquired using Keyence 3D (Keyence, Germany). Afterward, ImageJ software was used to measure the height and width of the droplets. The agar droplets were considered to have a shape of spherical cap, and their volume was calculated using the following equation^[^ ^27^ ^]^

(6) $$V=1/24\cdot \pi \cdot h(3d^{2}+h^{2})$$

where *h* is the height of the agar droplet and *d* is the diameter of the bottom of the agar droplet.

##### Bacterial Culture

*Escherichia coli* (*E. coli*) DSM498 was used as a target bacterial strain in this study. *E. coli* was cultivated in LB medium at 37 °C.

Prior seeding *E. coli* DSM498 on SM‐DMA, it was cultured in LB medium overnight; then, OD was measured and adjusted to OD_600_ = 1 to obtain the bacterial suspension of 10^8^ CFU mL^−1^. Afterward, 2 μL of bacterial suspension was manually pipetted onto the agar droplets of SM‐DMA. The SM‐DMA was placed into the Petri dish with water pad and sealed with parafilm. These samples were later put into an incubator under 37 °C for overnight cultivation.

##### Colorimetry‐Based Estimation of Bacterial Growth on SM‐DMA

After overnight cultivation of the bacteria on SM‐DMA, 2 μL of cell counting Kit‐8 was manually pipetted on each of the agar spots. The SM‐DMA was then incubated at room temperature in a sterile environment for 1 h. Afterward, the digital image of SM‐DMA was acquired using CanoScan 8800 F scanner. ImageJ software was used to obtain the diagram of the RGB signals for each droplet using “color profiler” function. After selecting the region of interest which is the center of the agar droplet, the result will be shown separately in RGB channel signals. The signal from blue channel will be further calculated by Equation (3) and used for the drug–dose analysis.

Based on values obtained after analysis of colorimetric images of SM‐DMA, the dose–response fitting was done using the “Slogistic1‐fit” of OriginPro 2022b.

The MIC can be determined through the following two methods. Visual method: The MIC was identified as the lowest drug concentration in the agar droplet that induced a visible color change from transparent to yellow. Statistical method: The MIC was derived by determining the drug concentration corresponding to the IC90 value on the fitted Boltzmann curve. This is done by tracing the *x*‐axis value at the point where IC90 occurs on the curve.

##### Statistical Analysis

All experiments were conducted in triplicate (*n* = 3). Scanned SM‐DMA slides with colorimetric viability data were quantified using ImageJ by extracting the blue channel intensity for each droplet of agar and normalizing it to the controls (bacteria without antibiotic), expressed as mean ± SD. Dose–response curves were fitted using a Boltzmann sigmoidal function in OriginPro 2022b (OriginLab Corp., USA). The derived IC90 values were used to estimate the MIC.

## Conflict of Interest

The authors declare no conflict of interest.

## Data Availability

All data necessary to evaluate the conclusions are provided in the paper. Additional data available upon request or in the RADAR4KIT repository at https://doi.org/10.35097/dkekuatttn0b4q7z.

## References

[1] B. Aslam , W. Wang , M. I. Arshad , M. Khurshid , S. Muzammil , M. H. Rasool , M. A. Nisar , R. F. Alvi , M. A. Aslam , M. U. Qamar , M. K. F. Salamat , Z. Baloch , Infect. Drug Resist. 2018, 11, 1645. 30349322 10.2147/IDR.S173867 PMC6188119

[2] World Bank Group , Drug‐Resistant Infections: A Threat to Our Economic Future, World Bank, Washington, DC 2017.

[3] V. M. Vashishtha , Indian Pediatr 2010, 47, 505. 20622280 10.1007/s13312-010-0087-1

[4] J. G. Bartlett , D. N. Gilbert , B. Spellberg , Clin. Infect. Dis. 2013, 56, 1445. 23403172 10.1093/cid/cit070

[5] E. Y. Klein , T. P. Van Boeckel , E. M. Martinez , S. Pant , S. Gandra , S. A. Levin , H. Goossens , R. Laxminarayan , Proc. Natl. Acad. Sci. U.S.A. 2018, 115, E3463. 29581252 10.1073/pnas.1717295115 PMC5899442

[6] R. Mirzaei , P. Goodarzi , M. Asadi , A. Soltani , H. A. A. Aljanabi , A. S. Jeda , S. Dashtbin , S. Jalalifar , R. Mohammadzadeh , A. Teimoori , K. Tari , M. Salari , S. Ghiasvand , S. Kazemi , R. Yousefimashouf , H. Keyvani , S. Karampoor , IUBMB Life 2020, 72, 2097. 32770825 10.1002/iub.2356 PMC7436231

[7] F. C. Tenover , in Encyclopedia of Microbiology, Elsevier, Amsterdam, Netherlands 2009, pp. 67–77.

[8] V. Prakash , J. S. Lewis , J. H. Jorgensen , Antimicrob. Agents Chemother. 2008, 52, 4528. 18838599 10.1128/AAC.00904-08 PMC2592869

[9] Clinical and Laboratory Standards Institute (CLSI) , Performance Standards for Antimicrobial Susceptibility Testing, 35th ed. (M100), CLSI, Wayne, PA 2025.

[10] T. T. N. Dung , V. V. Phat , C. Vinh , N. P. H. Lan , N. L. N. Phuong , T. Q. Le Ngan , G. Thwaites , L. Thwaites , M. Rabaa , A. T. K. Nguyen , P. T. Duy , BMC Infect. Dis. 2024, 24, 164. 38326753 10.1186/s12879-024-09028-2 PMC10848345

[11] M. M. Traczewski , E. Carretto , R. Canton , N. M. Moore , J. Clin. Microbiol. 2018, 56, e00272‐18. 29848561 10.1128/JCM.00272-18 PMC6062815

[12] S. A. Dunbar , Clin. Chim. Acta 2006, 363, 71. 16102740 10.1016/j.cccn.2005.06.023 PMC7124242

[13] I. Gajic , J. Kabic , D. Kekic , M. Jovicevic , M. Milenkovic , D. Mitic Culafic , A. Trudic , L. Ranin , N. Z. Opavski , Antibiotics (Basel) 2022, 11, 10427. 10.3390/antibiotics11040427 PMC9024665 35453179

[14] M. Balouiri , M. Sadiki , S. K. Ibnsouda , J. Pharm. Anal. 2016, 6, 71. 29403965 10.1016/j.jpha.2015.11.005 PMC5762448

[15] K. C. Carroll , B. D. Glanz , A. P. Borek , C. Burger , H. S. Bhally , S. Henciak , D. Flayhart , J. Clin. Microbiol. 2006, 44, 3506. 17021074 10.1128/JCM.00994-06 PMC1594749

[16] C. Alonso‐Tarrés , C. Benjumea Moreno , F. Navarro , A. C. Habison , E. Gonzàlez-Bertran , F. Blanco , J. Borràs , M. Garrigó , J. Saker , Eur. J. Clin. Microbiol. Infect. Dis. 2024, 43, 1533. 38825624 10.1007/s10096-024-04862-3 PMC11271345

[17] J. Q. Boedicker , L. Li , T. R. Kline , R. F. Ismagilov , Lab Chip 2008, 8, 1265. 18651067 10.1039/b804911d PMC2612531

[18] K. Churski , T. S. Kaminski , S. Jakiela , W. Kamysz , W. Baranska-Rybak , D. B. Weibel , P. Garstecki , Lab Chip 2012, 12, 1629. 22422170 10.1039/c2lc21284f

[19] M. Blom , T. L. Sørensen , F. Espersen , N. Frimodt‐Møller , Scand. J. Infect. Dis. 2002, 34, 430. 12160170 10.1080/00365540110080601

[20] A. Irvine , J. Watt , M. J. Kurth , J. V. Lamont , P. Fitzgerald , M. W. Ruddock , Res. Rep. Urol. 2024, 16, 327. 39619890 10.2147/RRU.S483147 PMC11608548

[21] J. K.‐H. Wat , M. Xu , L. Nan , H. Lin , K. K.-W. To , H. C. Shum , S. U. Hassan , Microsyst. Nanoeng. 2025, 11, 110. 40425590 10.1038/s41378-025-00938-y PMC12117033

[22] S. H. MacVane , H. P. Dwivedi , J. Antimicrob. Chemother. 2024, 79, i13. 39298359 10.1093/jac/dkae282 PMC11412245

[23] S. Mudenda , B. Chabalenge , V. Daka , R. L. Mfune , K. I. Salachi , S. Mohamed , W. Mufwambi , M. Kasanga , S. K. Matafwali , Pharm. Pract. (Granada) 2023, 14, 271.

[24] M. Breitfeld , C. L. Dietsche , M. A. Saucedo‐Espinosa , S. F. Berlanda , P. S. Dittrich , Small 2025, 21, e2410275. 39961047 10.1002/smll.202410275

[25] D. D. Kartsev , U. G. E. Joaquin , P. A. Anna , P. A. Levkin , Adv. Mater. Interfaces 2025, 12, 2400905.

[26] H. Tominaga , M. Ishiyama , F. Ohseto , K. Sasamoto , T. Hamamoto , K. Suzuki , M. Watanabe , Anal. Commun. 1999, 36, 47.

[27] A. D. Polipi︠a︡nin , A. V. Manzhirov , Handbook of Mathematics for Engineers and Scientists, Chapman & Hall/CRC, Boca Raton FL 2007.

[28] X. Yang , Y. Zhong , D. Wang , Z. Lu , Anal. Methods 2021, 13, 5211. 34694314 10.1039/d1ay01624e

[29] A. Zelazko , in Encyclopedia Britannica Online, Encyclopædia Britannica, Chicago, IL, October 22, 2025, https://www.britannica.com/science/RGB‐color‐model.

[30] EUCAST , Breakpoint Tables for Interpretation of MICs and Zone Diameters, Version 15.0, The European Committee on Antimicrobial Susceptibility Testing, Basel, Switzerland 2025.

[31] W. Feng , L. Li , E. Ueda , J. Li , S. Heißler , A. Welle , O. Trapp , P. A. Levkin , Adv. Mater. Interfaces 2014, 1, 1400269.

[32] A. A. Popova , K. Demir , T. G. Hartanto , E. Schmitt , P. A. Levkin , RSC Adv. 2016, 6, 38263.

[33] A. A. Popova , S. Dietrich , W. Huber , M. Reischl , R. Peravali , P. A. Levkin , SLAS Technol. 2021, 26, 274. 32791934 10.1177/2472630320934432

[34] A. A. Popova , M. Reischl , D. Kazenmaier , H. Cui , T. Amberger , P. A. Levkin , SLAS Technol. 2022, 27, 44. 35058192 10.1016/j.slast.2021.10.017

[35] Y. Tian , M. Seifermann , L. Bauer , C. Luchena , J. J. Wiedmann , S. Schmidt , A. Geisel , S. Afonin , J. Höpfner , M. Brehm , X. Liu , C. Hopf , A. A. Popova , P. A. Levkin , Small 2024, 20, e2307215. 38258390 10.1002/smll.202307215

[36] W. Lei , K. Demir , J. Overhage , M. Grunze , T. Schwartz , P. A. Levkin , Adv. Biosyst. 2020, 4, e2000073. 32875737 10.1002/adbi.202000073

[37] R. Strutt , B. Xiong , V. F. Abegg , P. S. Dittrich , Lab Chip 2024, 24, 1064. 38356285 10.1039/d3lc01024d PMC10898417

[38] M. Roca , M. Castillo , P. Marti , R. L. Althaus , M. P. Molina , J. Agric. Food Chem. 2010, 58, 5427. 20397732 10.1021/jf9040518

[39] M. S. Masoud , A. E. Ali , G. S. Elasala , Spectrochim. Acta, Part A, Mol. Biomol. Spectrosc. 2015, 149, 363. 10.1016/j.saa.2015.04.061 25974669

[40] M. Roca , L. Villegas , M. L. Kortabitarte , R. L. Althaus , M. P. Molina , J. Dairy Sci. 2011, 94, 1155. 21338781 10.3168/jds.2010-3599

[41] M. Huskey , P. Lewis , S. D. Brown , Hosp. Pharm. 2021, 56, 507. 34720153 10.1177/0018578720925389 PMC8554611

[42] Sigma‐Aldrich , Ampicillin Sodium Salt Product Information Sheet, https://www.sigmaaldrich.com/deepweb/assets/sigmaaldrich/product/documents/185/797/a2804pis.pdf (accessed: October 2025).

[43] S. R. Behin , I. S. Punitha , Int. J. Pharm. Sci. Rev. Res. 2012, 14, 22.

[44] V. Das Gupta , J. Pharm. Sci. 1984, 73, 565. 6327966 10.1002/jps.2600730434

[45] O. Svahn , E. Björklund , Int. J. Innovation Appl. Stud. 2015, 11, 872.

[46] J. Coates , B. R. Park , D. Le , E. Şimşek , W. Chaudhry , M. Kim , eLife 2018, 7, e32976. 29508699 10.7554/eLife.32976 PMC5847335

[47] N. Kadeřábková , A. J. S. Mahmood , D. A. I. Mavridou , npj Antimicrob. Resist. 2024, 2, 37. 39843555 10.1038/s44259-024-00051-6 PMC11721449

[48] J. W. Mouton , J. Meletiadis , A. Voss , J. Turnidge , J. Antimicrob. Chemother. 2018, 73, 2374. 30137390 10.1093/jac/dky232

[49] Aquarray , Product Catalogue 2023, Valid from: July 2023, p. 3.

